# Supplementary material for: Empirical methods for controlling false positives and estimating confidence in ChIP-Seq peaks
Source: BMC Bioinformatics. 2008 Dec 5;9:523. doi: 10.1186/1471-2105-9-523 (PMC2628906; doi:10.1186/1471-2105-9-523)
Supplement: Additional File 1 — A variety of html documents from the USeq web site detailing the available applications, their best usage, output file type descriptions, command line menus, etc. [file 1471-2105-9-523-S1.zip › USeqUserGuides/JavaDoc.zip!/JavaDoc/util/bio/cluster/package-tree.html]

util.bio.cluster Class Hierarchy


|  |  |  |
| --- | --- | --- |
| **Overview** \| **Package** \| Class \| Use \| **Tree** \| **Deprecated** \| **Index** \| **Help** | |  |
| **PREV**   **NEXT** | **FRAMES**    **NO FRAMES**     **All Classes** |  |


---


## Hierarchy For Package util.bio.cluster

**Package Hierarchies:**: All Packages

---

## Class Hierarchy

- java.lang.Object
  - util.bio.cluster.**Cluster** (implements java.io.Serializable)- java.awt.Component (implements java.awt.image.ImageObserver, java.awt.MenuContainer, java.io.Serializable)
      - java.awt.Container
        - javax.swing.JComponent (implements java.io.Serializable)
          - javax.swing.JPanel (implements javax.accessibility.Accessible)
            - util.bio.cluster.**ClusterDrawPanel**
          - java.awt.Window (implements javax.accessibility.Accessible)
            - java.awt.Frame (implements java.awt.MenuContainer)
              - javax.swing.JFrame (implements javax.accessibility.Accessible, javax.swing.RootPaneContainer, javax.swing.WindowConstants)
                - util.bio.cluster.**ClusterDrawFrame**
      - util.bio.cluster.**HierarchicalClustering**

---


|  |  |  |
| --- | --- | --- |
| **Overview** \| **Package** \| Class \| Use \| **Tree** \| **Deprecated** \| **Index** \| **Help** | |  |
| **PREV**   **NEXT** | **FRAMES**    **NO FRAMES**     **All Classes** |  |


---
